# Optimal weighting factor design based on entropy technique in finite control set model predictive torque control for electric drive applications

**DOI:** 10.1038/s41598-024-63694-5

**Published:** 2024-06-04

**Authors:** Muhammad Bilal Shahid, Weidong Jin, Muhammad Abbas Abbasi, Lian Li, Akhtar Rasool, Abdul Rauf Bhatti, Abdulkerim Sherefa Hussen

**Affiliations:** 1 School of Electrical Engineering, Southwest Jiaotong University, Chengdu City, Sichuan Province China; 2 China-ASEAN International Joint Laboratory of Integrated Transportation, Nanning University, Nanning, China; 3 Department of Electronic Engineering, Faculty of Engineering, The Islamia University, Bahawalpur, Pakistan; 4 Department of Electrical Engineering, Faculty of Engineering and Technology, University of Botswana, Gaborone, Botswana; 5 Department of Electrical Engineering and Technology, Government College University Faisalabad, Faisalabad, Pakistan; 6 Department of Electrical and Computer Engineering, Wolkite University, Wolkite, Ethiopia

**Keywords:** Model predictive torque control (MTPC), Weighting factor, Multi-decision making criteria, Entropy method, Induction motor, Electrical and electronic engineering, Engineering

## Abstract

In the conventional finite control set model predictive torque control, the cost function consists of different control objectives with varying units of measurements. Due to presence of diverse variables in cost function, weighting factors are used to set the relative importance of these objectives. However, selection of these weighting factors in predictive control of electric drives and power converters still remains an open research challenge. Improper selection of weighting factors can lead to deterioration of the controller performance. This work proposes a novel weighting factor tuning method based on the Multi-Criteria-Decision-Making (MCDM) technique called the Entropy method. This technique has several advantages for multi-objective problem optimization. It provides a quantitive approach and incorporates uncertainties and adaptability to assess the relative importance of different criteria or objectives. This technique performs the online tuning of the weighting factor by forming a data set of the control objectives, i.e., electromagnetic torque and stator flux magnitude. After obtaining the error set of control variables, the objective matrix is normalized, and the entropy technique is applied to design the corresponding weights. An experimental setup based on the dSpace dS1104 controller is used to validate the effectiveness of the proposed method for a two-level, three-phase voltage source inverter (2L-3P) fed induction motor drive. The dynamic response of the proposed technique is compared with the previously proposed MCDM-based weighting factor tuning technique and conventional MPTC. The results reveal that the proposed method provides an improved dynamic response of the drive under changing operating conditions with a reduction of 28% in computational burden and 38% in total harmonic distortion, respectively.

## Introduction

Induction motors (IM) are the workhorses of industry due to their numerous advantages such as low cost, ruggedness, reliability, high efficiency, low energy consumption, and minimum maintenance requirement^1,2^. The performance of IM can be improved by incorporating efficient control techniques in which field-oriented control (FOC) and direct torque control (DTC) are highly mature and commonly used methods^3,4^.

Recently, finite control set model predictive torque control (FCS-MPTC) has gained popularity among researchers due to its numerous benefits including effective incorporation of non-linearities into controller model, multivariable formulation, and constraint handling among others^5–8^. Like DTC, FCS-MPTC does not require any switching table and its simple structure makes it easier to implement. The optimal switching state of the inverter is obtained by an optimization-based approach that uses the cost function. The cost function consists of errors of control variables that are the difference between reference values and their future values at the next sampling instant. Normally, flux and torque are selected as control variables in the cost function for FCS-MPTC^9^. A mathematical model of an induction motor is used to predict future values of the controlled variables using permissible switching states. The switching state that generates the minimum cost function is applied to a two-level voltage source inverter.

**Figure 1:**
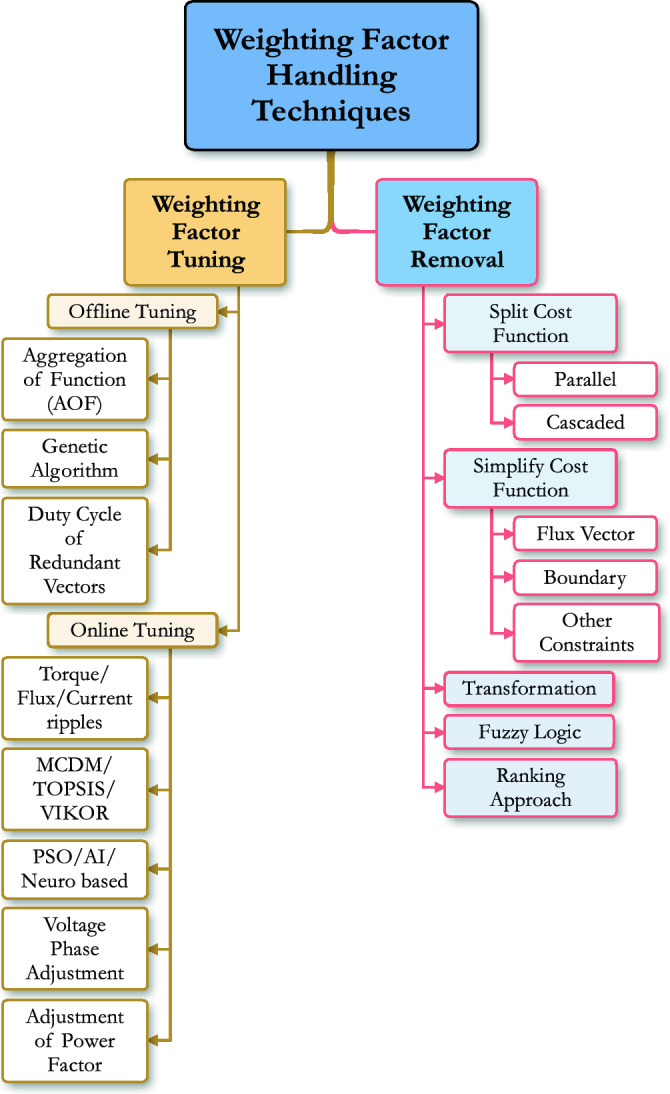
Different weighting factor removal and tuning techniques^14–24^.

Due to the presence of different variables with different units of measurements in a single cost function, it becomes challenging to adjust the relative importance (weights) of the controlled variables. This challenge is known as weighting factor design problem for FCS-MPTC^10,11^. Weighting factor designing methods can be broadly categorized into two groups: first is the weighting factor removal techniques, and the second is weighting factor tuning techniques. The weighting factor removal and tuning techniques can be further divided into various classes. A detailed classification of these methods is shown in Fig. 1. A detailed comparison of different available weighting factor methods in the literature is also given in Table 1. Many solutions to this problem have been proposed in literature based on offline tuning, weighting factor removal, and online tuning^12,13^. The offline tuning is simple but time-consuming; tedious work is required to tune the weighting factor and static weighting factor does not guarantee optimal performance on different operating points.

Another solution is to remove the weighting factor from cost function. Weighting factor removal methods include: a multi-ranking-based technique^25^, voltage tracking error method^26^, modulated technique^27^, and reference transformation^8,28^ . However, most of weighting factor removal methods increase the computational burden of the conventional FCS-MPTC^29^. In addition to these methods, weighting factor can be removed from the cost function by employing sequential or cascade MPTC. In^30^, a novel sequential model predictive torque control (SMPTC) methodology is presented for a six-phase induction machine, effectively eliminating the weighting factor requirement. The system employs a sequential framework to assess three distinct cost functions, each corresponding to a specific control purpose^31^. In^32^, a cascaded predictive control (CPC) approach was proposed for induction machine drives powered by a three-level NPC power converter.

**Table 1:** Summary of the PTC methods focused on weighting factor challenges.

PTC Methods	Limitations
Weighting factor removal by reference transformation^33,34^	Higher computational burden as compared with conventional PTC and difficult to incorporate multiple control objectives^35^
Weighting factor tuning based on coefficient of variation^36^	Optimized weights are uncertain in this method and complex calculations are required to implement on hardware
Weighting factor tuning based on TOPSIS and NSGA-II methods^37^	TOPSIS and NSGA-II algorithms require complex calculations leading to computational challenges^12^
Weighting factor removal by Ranking method^38^	Ranking based techniques become unfeasible as number of control objectives increases^39^
Tuning of weighting factor based on simple additive technique^40^	Although technique is simple but not suitable for multiple control objectives^11^
Weighting factor tuning based on current ripples^41^	Highly dependent on parameter estimation^8,42^
Tuning of weighting factor based on error of control objectives^43^	This method becomes challenging and complex when number of control objectives increases^44^
Weighting factor tuning using Genetic Algorithm (GA)^45^, Simulated Annealing (SA)^42^ or Gravitational Search Algorithm (GSA)^43^, Artificial Neural Network^46^, Ant colony based optimization^47^	These algorithms are very complex and pose computational challenges^48^
Weighting factor tuning based on algebraic/numerical techniques^49^	Design complexity increases as slection of weighting factor increases^50^
Weighting factor selection based on homogeneous cost functions^51–53^	This technique is relatively efficient but unable to include multiple control objectives^54^
Direct vector selection based techniques to remove weighting factors from cost function^55,56^	Direct vector selection techniques provid lower computational burden and lower complexity , however cannot incorporate multiple control objecitve^57^
Weighting factor elimination by using cascaded structure of FCS-MPC^58,59^	The cascaded structure highly depended on proper selection of dealing cascaded structure^60^

This approach preserves the advantageous qualities such as, robustness and control performance, of the classic model predictive torque control (MPTC)^11,61^. However, removing the weighting factor involves the formulation and ranking of multiple objective functions which complicates the algorithm and increases the computational burden. Online weighting factor techniques change the weights of corresponding control objectives according to operating conditions to optimize the performance. These methods can be broadly categorized into (i) meta-heuristic tuning methods (ii) artificial neural networks (ANN) based methods (iii) Multi-Criteria Decision Making (MCDM) techniques and (iv) miscellaneous methods. Meta-heuristic techniques such as simulated annealing (SA) based tuning technique is proposed in^62^. However, online computing of weighting factor by such methods limits the search accuracy and increases the computational complexity^47^. Other meta-heuristic methods include gravitational search algorithm (GSA)^43^, non-dominated sorting genetic algorithm II (NSGA-II) and Particle Swarm Optimization (PSO)^10–12^ . ANN methods are reported in^46,63–66^. However, they also suffer from higher computational burden and tend to increase the complexity of the controller. Miscellaneous methods such as two-stage weighting factor tuning technique^67^ and equal-weighted weighting factor selection technique^68^ also tend to increase the computational cost of the control algorithms and are not feasible for practical implementation^47,69–73^. In comparison the these methods, some of the MCDM methods do not pose computational challenges and are ideally aligned for implementation on the modern hardware^39,74,75^. Although various methods have been proposed in the literature to deal with the weighting factor problem, the selection of the weighting factor is still an open challenge due to its computational burden and complexity. Therefore, this paper presents a novel method to optimise the tuning of the weighting factor. The proposed method is based on the entropy technique used to determine appropriate weights for the control variables used in the cost function. This technique is suitable for quantifying the uncertainty or disorder in a given data set (obtained from the error of control objectives). The entropy technique provides a balance between control objectives and assigns appropriate weights in the cost function according to relative significance. The main contributions of the proposed technique are as follows:

- The weighting factor is tuned online by employing a simple MCDM technique called entropy method therefore, no need to tune weighting factor.
- The proposed method demonstrates robustness against motor parameter variations and model mismatching.
- The switching frequency, THD and computational burden of the entropy based MPTC is lower when compared to other MPTC methods.

The paper is organized as follows: Section “Conventional model predictive torque control” describes the mathematical model of the induction motor. The general formulation of FCS-MPTC is presented in Section “VIKOR-MPTC”. The proposed tuning method is given in Section “Proposed weighting factor tuning based on entropy method”. In the next section comprehensive discussion of different results is provided.

### Dynamical model of induction motor and VSI

The modeling of squirrel cage IM and 2L-3P VSI is presented in this section. The standard equations of IM in stationary reference frame ($\alpha$-$\beta$ frame) can be expressed as^6,73,76,77^.

The voltage equations of stator and rotor can written as:

1 $$\begin{aligned}{} & {} \overrightarrow{v}_{\text{s}}=\text{R}_{\text{s}\quad }\overrightarrow{i_s} +\frac{\text{d}\overrightarrow{\psi _s}}{\text{dt}} \end{aligned}$$

2 $$\begin{aligned}{} & {} 0=\text{R}_{\text{r}}\overrightarrow{i_r}+\frac{\text{d}\overrightarrow{\psi _r}}{\text{dt}}-\text{j}\omega \overrightarrow{\psi _r} \end{aligned}$$

The flux equations of both stator and rotor are expressed as:

3 $$\begin{aligned} \overrightarrow{\psi _s}= & {} \text{L}_{\text{s}}\overrightarrow{i_s}+\text{L}_{\text{m}}\overrightarrow{i_r} \end{aligned}$$

4 $$\begin{aligned} \overrightarrow{\psi _r}= & {} \text{L}_{\text{r}}\overrightarrow{i_r}+\text{L}_{\text{m}}\overrightarrow{i_s} \end{aligned}$$

The electromagnetic torque generated by the motor is given by:

5 $$\begin{aligned} \text{T}=\frac{2}{3}\text{p}\mathfrak {Im}\{{\overrightarrow{\bar{\psi _s}}}\overrightarrow{ i_s}\} =\frac{2}{3}\text{p}\mathfrak {Im}\{{\overrightarrow{\bar{\psi _r}}\overrightarrow{i_r}}\} \end{aligned}$$

**Figure 2:**
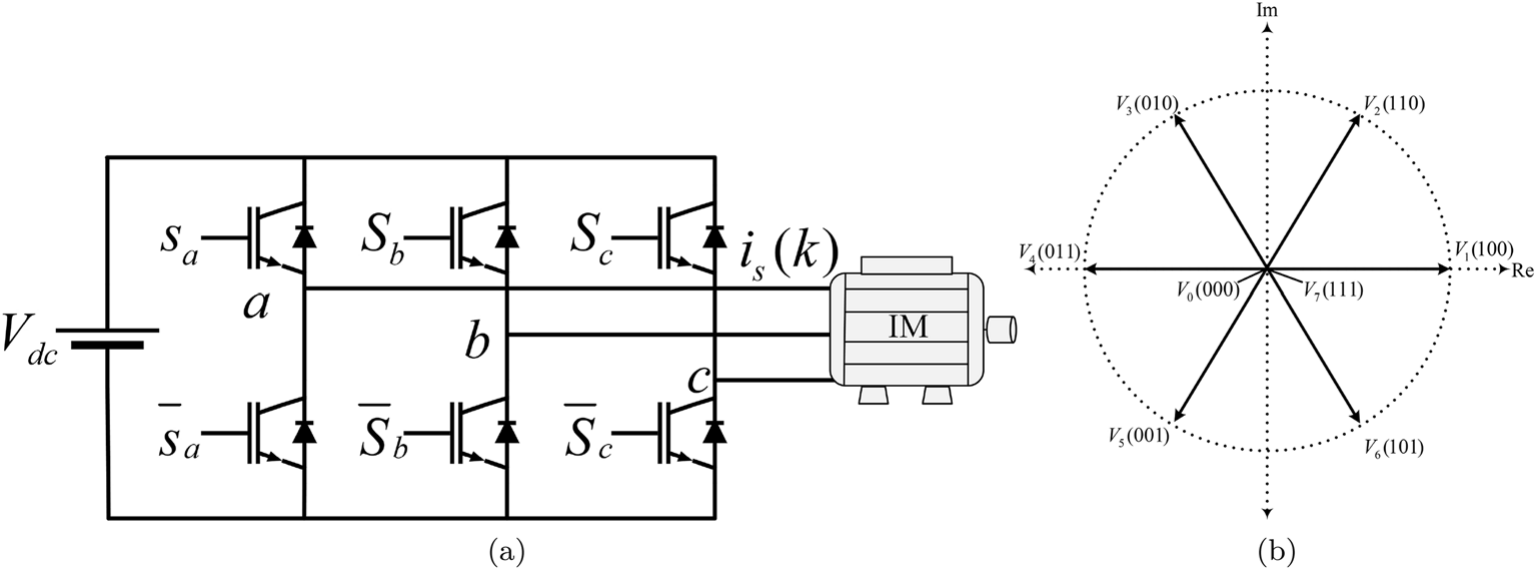
Two level three phase voltage source inverter. (**a**) Inverter topology, (**b**) Inverter possible switching vectors.

Finally, the mechanical equation of motor can be written as:

6 $$\begin{aligned} \text{J}\frac{\text{d}\omega _{\text{m}}{\text{dt}}}{+}\text{B}\omega _{\text{m}}=\text{T}-\text{T}_{\text{l}} \end{aligned}$$

where the subscript $\text{s}$, $\text{r}$ represents the stator and rotor variables respectively; $\text{R}$, ${\overrightarrow{i}}$, and ${\overrightarrow{\psi }}$ are resistance, current, and flux; $\overrightarrow{\text{v}}_{\text{s}}=\text{v}_{\text{s}\alpha }+\text{jv}_{\text{s}\beta }$ is the voltage vector, $\overrightarrow{i_s}=\text{i}_{\text{s}\alpha }+\text{ji}_{\text{s}\beta }$, $\overrightarrow{\psi _s}=\psi _{\text{s}\alpha }+\text{j}\psi _{\text{s}\beta }$, and $\overrightarrow{\psi _r}=\psi _{\text{r}\alpha }+\text{j}\psi _{\text{r}\beta }$ are the stator current, rotor current vector, stator flux, and rotor flux vector; $\text{R}_{\text{s}}$, $\text{R}_{\text{r}, \text{L}_{\text{s}}}$, $\text{L}_{\text{r}}$, and $\text{L}_{\text{m}}$ are the stator resistance, rotor resistance, stator inductance, rotor inductance, and mutual inductance; $\omega _{\text{m}}$, $\text{T}$, $\text{T}_{\text{l}}$, $\text{J}$, and $\text{P}$ are the mechanical speed, electromagnetic torque, load torque, total moment of inertia of the system; $\mathfrak {Im}$ represents the imaginary part of the complex variables. The model can be represented in state-space by selecting stator current ${\overrightarrow{i_s}}$, and rotor flux ${\overrightarrow{\psi _r}}$ as state variables.

7 $$\begin{aligned} \frac{\text{d}\overrightarrow{i_s}}{\text{dt}}= & {} \frac{-1}{\tau _\sigma }\overrightarrow{i_s} +\frac{\text{k}_{\text{r}}}{\text{R}_\sigma \tau _\sigma } \left( \frac{1}{\tau _{\text{r}}-\text{j}\omega }\right) \overrightarrow{\psi _r}+\frac{1}{\text{R}_\sigma \tau _\sigma }\text{v}_{\text{s}} \end{aligned}$$

8 $$\begin{aligned} \frac{\text{d}\overrightarrow{\psi _r}}{\text{dt}}= & {} \frac{{sps}_{\text{m}}}{\tau _{\text{r}}} \overrightarrow{i_s}-\left( \frac{1}{\tau _{\text{r}}}-\text{j}\omega \right) \overrightarrow{\psi _r} \end{aligned}$$

where $\text{k}_{\text{r}}=\frac{\text{L}_{\text{m}}}{\text{L}_{\text{r}}}$, $\tau _{\text{r}}=\frac{\text{L}_{\text{r}}}{\text{R}_{\text{r}}}$, are the rotor coupling coefficient and rotor time constant respectively; $\tau _{\sigma } = \frac{{\left( {L_{s} L_{r} - L_{m}^{2} } \right)}}{{L_{r} \left( {R_{s} + R_{r} k_{r}^{2} } \right)}}$ is the stator transient time constant. A final discrete model can be obtained by discretizing (7) and (8) using Euler’s first-order discretization method. The IM motor is fed by two level three phase voltage source inverter (VSI) which is shown in Fig. 2. The VSI is energized by a constant (dc) voltage source $\text{V}_{\text{dc}}$. The switches $\text{S}_{\text{a}}$, $\text{S}_{\text{b}}$, $\text{S}_{\text{c}}$, $\bar{\text{S}_{\text{a}}}$, $\bar{\text{S}}_{\text{b}}$, and $\bar{\text{S}}_{\text{c}}$ are operated in complementary fashion to avoid “shoot-through” faults which may short-circuit the terminals of the DC source. In the direct switching method, there are eight switching states or voltage vectors (VVs) $\text{v}_{\text{n}}=\{\text{v}_0,\text{v}_1,\ldots \text{v}_7\}$ as depicted in Table 2.

**Table 2:** Voltage vectors of 2L-VSI.

$\text{v}_{\text{n}}$	$\text{S}=[\text{S}_{\text{a}}\text{S}_{\text{b}}\text{S}_{\text{c}}]$	$\overrightarrow{v}=\text{v}_\alpha +\text{jv}_\beta$
$\text{v}_0$	$\text{000}$	$\text{0}$
$\text{v}_1$	$\text{100}$	$\frac {2}{3}\text{V}_{\text{dc}}$
$\text{v}_2$	$\text{110}$	$\text{V}_{\text{dc}}(\frac {1}{3}+\text{j}\frac {\sqrt{3}}{3})$
$\text{v}_3$	$\text{010}$	$\mathrm {V_{dc}(-\frac {1}{3}+\text{j}\frac {\sqrt{3}}{3})}$
$\text{v}_4$	$\text{011}$	$-\frac {2}{3}\text{V}_{\text{dc}}$
$\text{v}_5$	$\text{001}$	$\text{V}_{\text{dc}}(-\frac {1}{3}-\text{j}\frac {\sqrt{3}}{3})$
$\text{v}_6$	$\text{101}$	$\text{V}_{\text{dc}}(\frac {1}{3}-\text{j}\frac {\sqrt{3}}{3})$
$\text{v}_7$	$\text{111}$	$\text{0}$

## Conventional model predictive torque control

**Figure 3:**
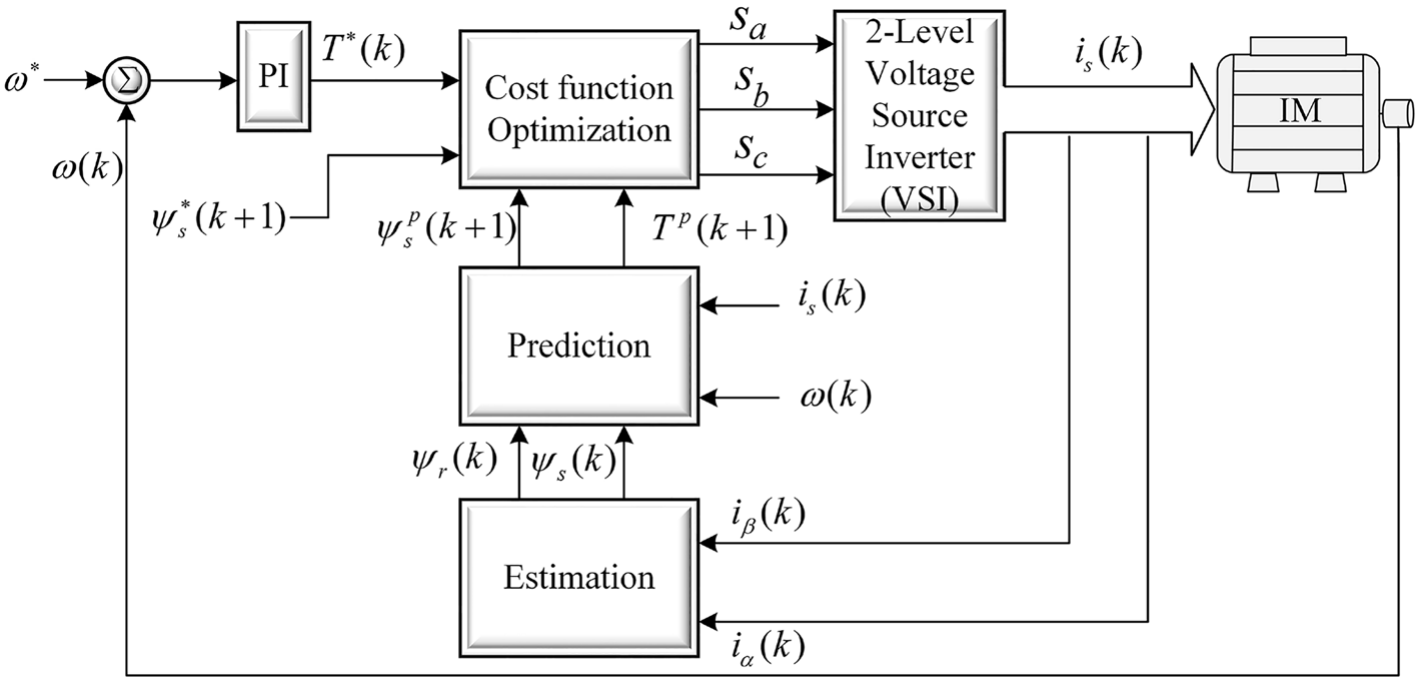
Conventional predictive torque control for induction motor drive fed by two level voltage source inverter.

The structure of conventional FCS-MPTC is shown in Fig. 3. It can be observed from the figure that FCS-MPTC consists of two loops: an outer loop for speed regulation and an inner torque control loop. The speed loop generates a torque reference signal with the help of a PI controller. Flux reference is kept constant to the nominal value since this work does not consider efficiency optimization and field weakening. The inner FCS-MPTC algorithm works in three main steps namely (i) estimation of controlled variables that cannot be measured, (ii) prediction of controlled variables from estimated and measured variables (iii) cost function optimization^77,78^. The mathematical details of these steps are provided next.

For flux and torque estimation, a simple current model (CM) estimator^76^ is used in this work. Stator estimation can be obtained by discretizing and rearranging (1) as:

9 $$\begin{aligned} \overrightarrow{\hat{\psi }_s}(\text{k})=\overrightarrow{\hat{\psi }_s} (\text{k}-1)+\text{T}_{\text{s}}\text{v}_{\text{s}}-\text{T}_{\text{s}} \text{R}_{\text{s}}\overrightarrow{i_s}(\text{k}) \end{aligned}$$

FCS-MPTC is required to predict the stator flux and electromagnetic torque based on the estimation of flux. By using (9), the prediction of stator flux at an instant of $\mathrm {(k+1)}$ can be obtained as:

10 $$\begin{aligned} \overrightarrow{\psi ^p_s}(\text{k}+1)=\overrightarrow{\psi ^p_s}+\text{T}_{\text{s}} \text{v}_{\text{s}}-\text{T}_{\text{s}}\text{R}_{\text{s}}\overrightarrow{i_s}(\text{k}) \end{aligned}$$

Electromagnetic torque can be predicted by using predicted current and estimated flux.

11 $$\begin{aligned} \text{T}^{\text{p}}(\text{k}+1)=\frac{2}{3}\text{p}\mathfrak {Im} \overrightarrow{\psi ^p_s}(\text{k}+1)\overrightarrow{i^p_s}(\text{k}+1) \end{aligned}$$

where is current prediction equation is given as follows:

12 $$\begin{aligned} \overrightarrow{i_s^p}(\text{k}+1) =\left( 1+\frac{\text{T}_{\text{s}}}{\tau _\sigma }\right) \overrightarrow{i_s}(\text{k}) +\frac{\text{T}_{\text{s}}}{\text{R}_\sigma \left( \text{T}_{\text{s}}+\tau _\sigma \right) }\Bigl \{\left( \frac{\text{k}_{\text{r}}}{\tau _{\text{r}}}-\text{jk}_{\text{r}}\omega \right) \overrightarrow{\hat{\psi }_r}(\text{k}) +\text{v}_{\text{s}}(\text{k})\Bigl \} \end{aligned}$$

Finally, the cost function is used to minimize the error in controlled variables and to determine optimal voltage vector. The cost function is formulated by adding torque and flux errors i.e. $\mathrm {T^*-T(k+1)}$ and $\mathrm {(|\overrightarrow{\psi _s}^*|-|\overrightarrow{\psi _s}(k+1)|)}$.

13 $$\begin{aligned} \text{g}=|\text{T}^*-\text{T}^{\text{p}}(\text{k}+1)| +\text{w}|\overrightarrow{\psi _s}^*-\overrightarrow{\psi _s}^{\text{p}}(\text{k}+1)| \end{aligned}$$

where $\text{w}$ is the weighting factor that defines the relative importance of control objectives. The cost function is evaluated for all the admissible voltage vectors for 2L-VSI (six active and two null VVs) at each sampling instant. The voltage vector that minimizes the value of the cost function is chosen and applied to VSI. In conventional FCS-MPTC, the nominal value of the weighting factor is defined as:

14 $$\begin{aligned} \text{w}=\frac{\text{T}_{\text{norm}}}{\psi _{\text{norm}}} \end{aligned}$$

## VIKOR-MPTC

The VlseKriterijumska Optimizacija I Kompromisno Resenje (VIKOR) approach was developed for the multi-criteria optimization problems in complex systems^79^. The details of this method applied to two level three phase VSI for induction motors are given in^48^ . The VIKOR approach can be categorized into following sequential steps:

1. Dataset Generation
2. Identification of optimal and suboptimal solutions
3. Utility and Regret measure computation
4. VIKOR index calculation

In order to provide a dataset that is appropriate for the proposed technique, the single cost-function utilized in the conventional PTC is partitioned into separate cost functions for torque and stator flux.

15 $$\begin{aligned} (\text{x}_{\text{T}})_{\text{m}}= & {} |\text{T}_{\text{e}}^*-\text{T}_{\text{e}}(\text{k}+1)_{\text{m}}| \end{aligned}$$

16 $$\begin{aligned} (\text{x}_\psi )_{\text{m}}= & {} |\psi _{\text{s}}^*-\psi _{\text{s}}(\text{k}+1)_{\text{m}}| \end{aligned}$$

where $\text{x}_{\text{T}}$ and $\text{x}_\psi$ are torque error cost function and flux error based cost function respectively. The above cost functions are evaluated for all the admissible voltage vectors and the resulting values are expressed in the form of following performance data matrix ${\varvec{X_{ij}}}$.

17 $$\begin{aligned} {\varvec{X_{ij}}}= \begin{bmatrix} (\text{x}_{\text{T}})_{\text{s0}} &{} (\text{x}_\psi )_{\text{s0}}\\ (\text{x}_{\text{T}})_{\text{s1}} &{} (\text{x}_\psi )_{\text{s1}} \\ \vdots &{} \vdots \\ (\text{x}_{\text{T}})_{\text{s6}} &{} (\text{x}_\psi )_{\text{s6}}\\ \end{bmatrix} \end{aligned}$$

The following expression can be applied to the generated data set to determine the ideal solution and non-ideal solution $\mathrm {(Y^+, Y^-)}$ for each control objective. These solutions simply represent maximum and minimum values of torque and flux errors in single sampling interval.

18 $$\begin{aligned} \text{Y}^+= & {} \Bigl \{(\text{maxX}_{\text{ij}}|\text{j}\in \text{J}) \; \text{or} \;(\text{minX}_{\text{ij}}|\text{j}\in {\bar{\text{J}}})\Big |_{\text{i}=1,2,\ldots 7}\Bigl \} \end{aligned}$$

19 $$\begin{aligned} \text{Y}^-= & {} \Bigl \{(\text{minX}_{\text{ij}}|\text{j}\in \text{J}) \; \text{or} \; (\text{maxX}_{\text{ij}}|\text{j}\in {\bar{\text{J}}})\Big |_{\text{i}=1,2,\ldots 7}\Bigl \} \end{aligned}$$

where

20 $$\begin{aligned} \text{J}=\Bigl \{\text{j}= 1,2,\ldots \text{n}\Big |\text{X}_{\text{ij}},\; \; \text{for} \; \text{benefit} \; \text{criteria} \Bigl \} \end{aligned}$$

21 $$\begin{aligned} {\bar{\text{J}}}=\Bigl \{ \text{j}= 1,2,\ldots \text{n}\Big |\text{X}_{\text{ij}},\; \; \text{for} \; \text{cost} \; \text{criteria} \Bigl \} \end{aligned}$$

where (20) represents the maximum value for optimal performance and (21) represents objectives where lower values are desirable to meet certain constraints or to minimize undesirable effects. The utility $U_i$ and regret $R_i$, represents the average and worst scores respectively. $U_i$ and $R_i$ can be obtain from:

22 $$\begin{aligned} \text{U}_{\text{i}}= & {} \sum _{\text{j}=1}^{\text{n}} \text{w}_{\text{j}} \frac{\text{X}^*-\text{X}_{\text{ij}}}{\text{X}^*-{\bar{\text{X}}}_{\text{j}}} \end{aligned}$$

23 $$\begin{aligned} \text{R}_{\text{i}}=\text{max}|_{\text{j}} \biggr [\text{w}_{\text{j}}\frac{\text{X}^*-\text{X}_{\text{ij}}}{\text{X}^*-{\bar{\text{X}}}_{\text{j}}}\biggr ] \end{aligned}$$

where $\text{w}_{\text{j}}$ represents the weighting of *j*th iteration. In Vik-MPTC weights are selected on given relation,

24 $$\begin{aligned} \sum _{\text{j}=1}^{\text{n}}\text{w}_{\text{j}}=1 \end{aligned}$$

The VIKOR index, denoted as $Q_i$, is utilized for the purpose of identifying the most optimal selection among the options that are now available. This index can be mathematically represented in the following manner.

25 $$\begin{aligned} \text{Q}_{\text{i}}=\text{v}\biggr [\frac{\text{U}_{\text{i}}-\text{U}^*}{\bar{\text{U}}-\text{U}^*}\biggr ]+(1-\text{v})\biggr [\frac{\text{R}_{\text{i}}-\text{R}^*}{\bar{\text{R}}-\text{R}^*} \biggr ] \end{aligned}$$

where $\text{U}^*=\text{min}(\text{U}_{\text{i}})$, ${\bar{\text{U}}}=\text{max}(\text{U}_{\text{i}})$, $\text{R}^*=\text{min}(\text{R}_{\text{i}})$, $\bar{\text{R}}=\text{max}(\text{R}_{\text{i}})$, and $\text{v}$ is the group utility factor normally set to 0.5^80^.

## Proposed weighting factor tuning based on entropy method

The proposed method is based on well known multi-criteria decision-making (MCDM) algorithm called entropy method^75,81^. It is based on the notion of information entropy that measures the uncertainty of a system. In the context of FCS-MPTC, entropy is used to measure the degree of heterogeneity among the controlled variables. The weighting factor tuning is also based on the measure of this heterogeneity level. The weight is adjusted in such a way that highly diverse criteria gets equally importance whereas in similar criteria one variable may get different importance than the other variables.

In the first step, a data set is obtained based on available alternatives and required criteria. After that, the data set is normalized to a scale of zero to one. In the proposed work, the data set consists of torque and flux errors as follows.

26 $$\begin{aligned} \varvec{(g_T)}_{\text{n}}=|\text{T}_{\text{e}}^*-\text{T}_{\text{e}}(\text{k}+1)_{\text{n}}| \end{aligned}$$

27 $$\begin{aligned} \varvec{(g_\psi )_n}=|\psi _{\text{s}}^*-\psi _{\text{s}}(\text{k}+1)_{\text{n}}| \end{aligned}$$

The data set is formed by torque and flux errors as expressed in

28 $$\begin{aligned} \varvec{X}=[(\text{g}_{\text{T}})_{\text{n}} \; \; \; \;(\text{g}_\psi )_{\text{n}}] \end{aligned}$$

where ${\varvec{X}}$ data set contains different magnitudes and units of the control variables. In the next step, normalization is obtained for the data set by using the following expression.

29 $$\begin{aligned} \varvec{N_{ij}}=\frac{\text{X}_{\text{ij}}}{\sum _{\text{i}=1}^{\text{n}} \text{X}_{\text{ij}}} \; \; \; \; \; \; \; \text{i}=1,2,\ldots 7 \end{aligned}$$

In the next step entropy is obtained by using expression (29) on a normalized data set.

30 $$\begin{aligned} \varvec{E_j}=-\frac{1}{\ln (\text{m})}{\sum _{\text{i}=1}^{\text{m}} \varvec{N_{ij}}\ln (\varvec{N_{ij}})} \end{aligned}$$

where $\text{m}$ is the number of control objective. The deviation rate of the degree of entropy is given as:

31 $$\begin{aligned} \varvec{d_j}=1-\text{E}_{\text{j}} \; \; \; \; \; \; \; \text{j}=1,2,\ldots \text{m} \end{aligned}$$

Finally, entropy weight can be obtained by using:

32 $$\begin{aligned} \varvec{w_j}=\frac{\text{d}_{\text{j}}}{\sum _{\text{i}=1}^{\text{n}}\text{d}_{\text{j}}} \end{aligned}$$

where $\text{j}$ is the number of control objectives and in this work it is equal to 2. The weights are denoted as $w_1$ and $w_2$ for torque and flux, respectively. The obtained weighting factors will be used in the following cost function.

33 $$\begin{aligned} \text{C}=\text{w}_1|\text{T}_{\text{e}}^*-\text{T}_{\text{e}}(\text{k}+1)_{\text{m}}| +\text{w}_2|\psi _{\text{s}}^*-\psi _{\text{s}}(\text{k}+1)_{\text{m}}| \end{aligned}$$

To explain the working of the method, an example is presented here. The torque and flux errors used in this example are randomly taken from a single sampling instant under steady state condition.

34 $$\begin{aligned} \varvec{g_T}= & {} [1.6800\; \; 0.9950\; \; 1.7330\; \; 3.0950 \; \; 3.2490\; \; 2.1030\; \; 1.0270] \end{aligned}$$

35 $$\begin{aligned} \varvec{g_\psi }= & {} [0.0047\; \; 0.0042\; \; 0.0253\; \; 0.0157\; \; 0.0145\; \; 0.0347\; \; 0.0256] \end{aligned}$$

A characteristic matrix is formed from above errors as:

36 $$\begin{aligned} {\varvec{X}}=\begin{bmatrix} {1.6800} &{} {0.0047} \\ {0.9950} &{} {0.0042} \\ {1.7330} &{} {0.0253} \\ {3.0950} &{} {0.0157} \\ {3.2490} &{} {0.0145} \\ {2.1030} &{} {0.0347} \\ {1.0270} &{} {0.0256} \end{bmatrix} \end{aligned}$$

The data set is normalized by using (29):

37 $$\begin{aligned} {\varvec{N}=}\begin{bmatrix} {0.1210} &{} {0.0374} \\ {0.0717} &{} {0.0337} \\ {0.1248} &{} {0.2031}s \\ {0.2230} &{} {0.1260} \\ {0.2340} &{} {0.1162} \\ {0.1515} &{} {0.2783} \\ {0.0740} &{} {0.2054} \end{bmatrix} \end{aligned}$$

The degree of entropy, deviation of entropy, and entropy weight can be obtained using (30), (31), and (32):

38 $$\begin{aligned} \varvec{E_j}= & {} [0.8932 \; \; \; \;0.8430] \end{aligned}$$

39 $$\begin{aligned} \varvec{d_j}= & {} [0.1068 \; \; \; \;0.1570] \end{aligned}$$

40 $$\begin{aligned} \varvec{w_j}= & {} [0.4050 \; \; \; \;0.5950] \end{aligned}$$

**Figure 4:**
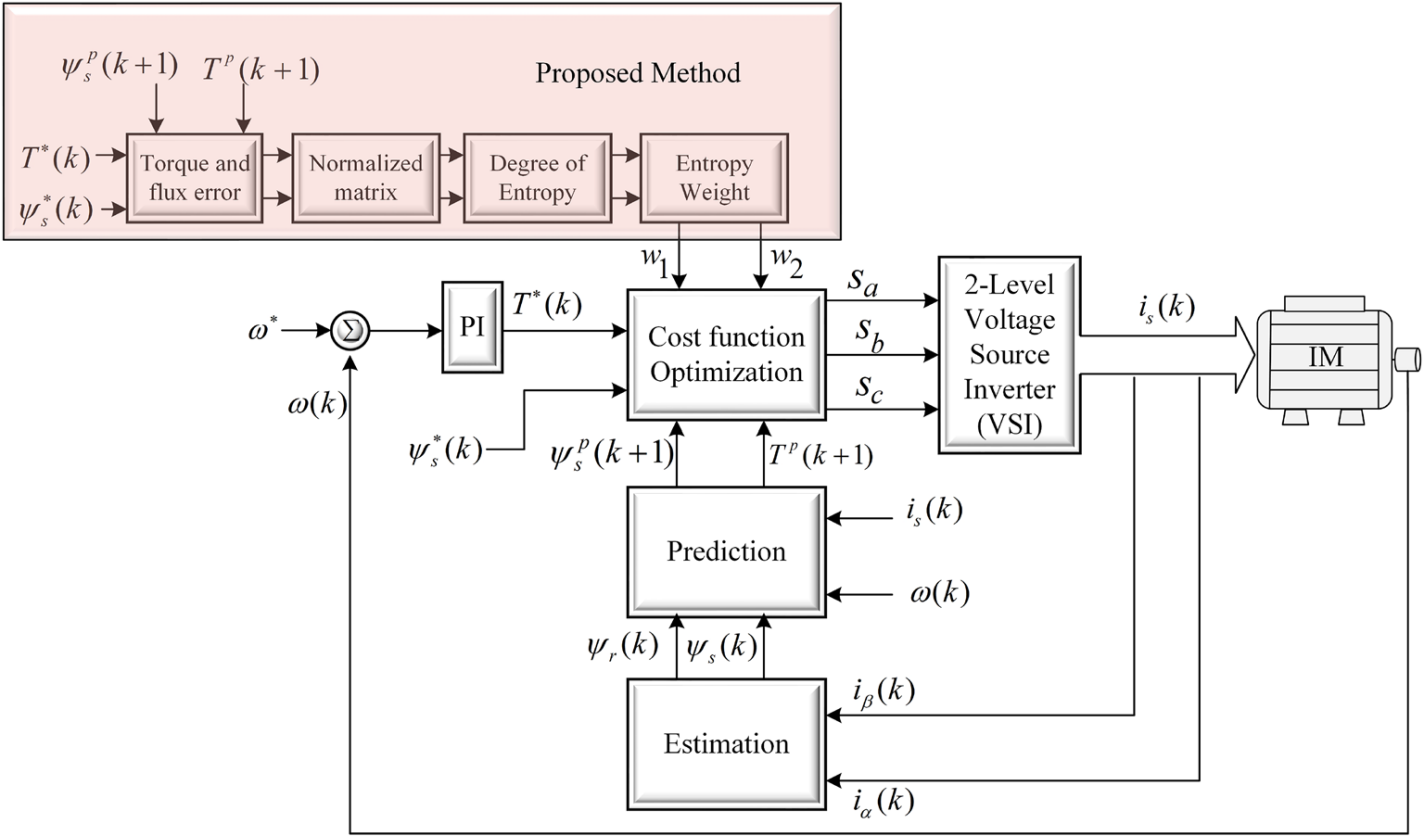
Proposed weighting factor selection method based on entropy technique.

The complete block diagram of the proposed method is shown in Fig. 4.

The figure shows that the system consists of main blocks of predictive control. The proposed method for tuning of weighting factor works in the following four steps:

i. Obtaining error data of the control variable (26), (27), and (28)
ii. Normalizing the error data because of different variables (29)
iii. The deviation rate of the degree of entropy obtained (30) and (31)
iv. Entropy weight is obtained to fit in the cost function (32)

**Figure 5:**
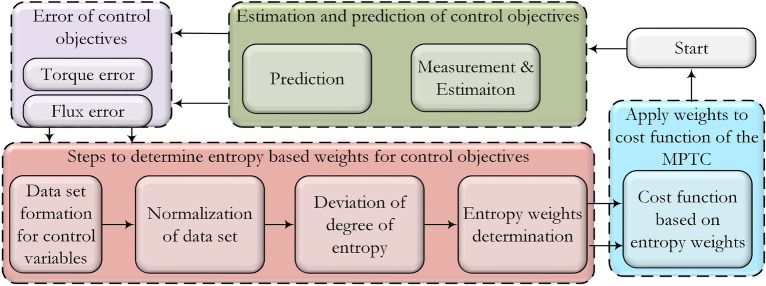
Flow chart diagram of entropy based weighting factor tuning.

These steps are also summarized in a block diagram in Fig. 4. The flowchart of the proposed method is shown in Fig. 5. The proposed method consists of the following phases that are error calculation, formation of data set by using errors of control objectives, normalization of the data set, derivation of the degree of entropy, and determination of weights

## Experimental results

**Figure 6:**
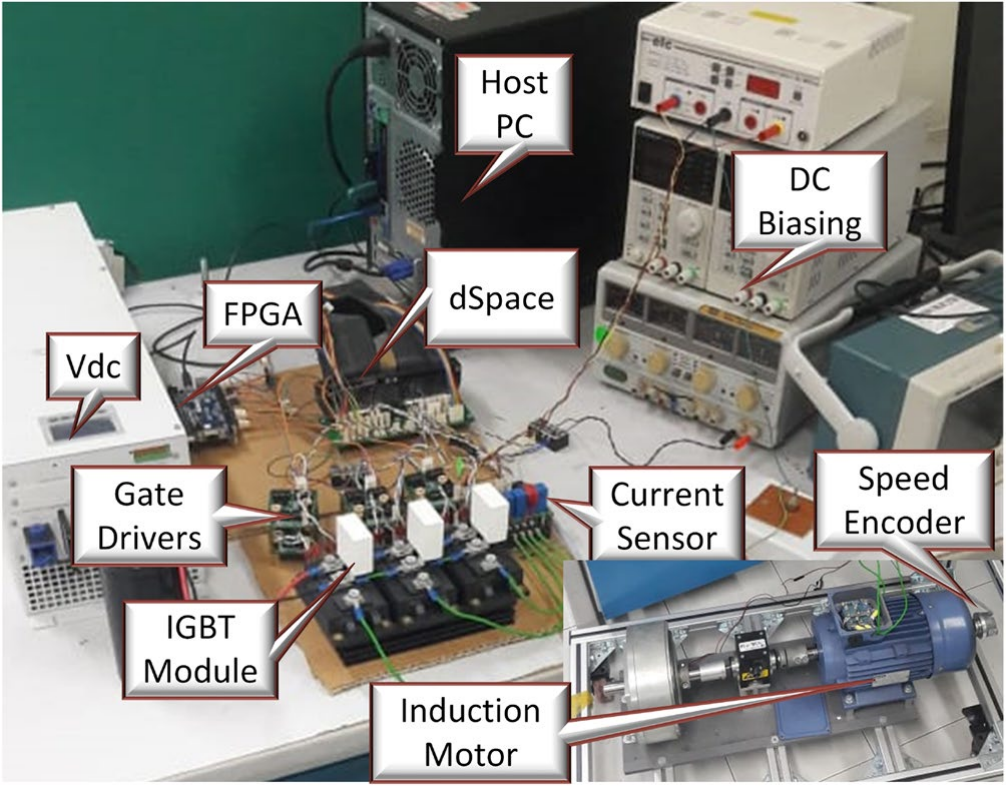
Experimental setup.

The experimental test setup depicted in Fig. 6, serves as a means of validating the efficacy of the proposed methodology. The apparatus comprises a dSpace DS1104 controller board, an FPGA board, a speed encoder, an IGBT module, gate drivers, a DC voltage source, and an induction motor. The controller board comprises an ADC, a DAC, a DSP floating point processor, and an incremental encoder. Programming of the controller is performed using the C programming language, the dSpace controller function library, and associated software to attain the desired results. The FPGA board generates the blanking time for the IGBTs and gate driver circuit. The speed encoder and current sensor are used to measure the speed of the induction motor and its current. A hysteresis brake is utilized in conjunction with the motor to serve as a load, with a proportional amplifier controlling the brake. The control algorithm comprises an outer speed load and an inner torque loop; therefore, two different timers are employed with different sampling times. The newly proposed weighting factor selection technique (Ent-MPTC) is compared with conventional model predictive torque control (Con-MPTC)^82^ and VIKOR-based weighting factor technique (Vik-MPTC)^48,80,83^

The parameters of the induction motor and controller are given in Table 3. The experiments have been performed on a sampling time of $\mathrm {60\, \upmu sec}$, and the drive’s performance is evaluated under various tests, and the corresponding results are given here.

**Table 3:** Motor and controller parameters.

Parameters	Value	Parameters	Value
Rated torque, $T_{norm}$	$10\,\text{Nm}$	Base speed, $\omega _{\text{base}}$	$120\,\mathrm {rad/sec}$
Stator resistance, $\text{R}_{\text{s}}$	${3\,\Omega }$	Frequency, $\text{f}$	$50\,\text{Hz}$
Stator inductance, $\text{L}_{\text{s}}$	$342\,\text{mH}$	Total viscous friction, $\text{B}$	$0.0042\,\mathrm {N\,m\,sec}$
Rotor resistance, $\text{R}_{\text{r}}$	$4\,\Omega$	Motor power, $\text{P}$	$1.5\,\text{kW}$
Rotor inductance, $\text{L}_{\text{r}}$	$351\, \text{mH}$	Nominal weighting factor, $\lambda _{\text{norm}}$	10.53
Mutual inductance, $\text{L}_{\text{m}}$	$324\, \text{mH}$	Torque loop sampling time, $\text{T}_{\text{s}}$	$60\,\upmu {\text{sec}}$
Total inertia, $\text{J}$	$0.01178\, \mathrm {kg\,m}^2$	Speed loop sampling time, $\mathrm {T_s}$	$\mathrm {4\, msec}$
DC source, $\text{v}_{\text{dc}}$	$460\,\text{V}$	No. of pole pairs, $\text{p}$	$\text{2}$
Rated flux, $\psi _{\text{norm}}$	$0.9\,\text{Wb}$		

### Transient response

**Figure 7:**
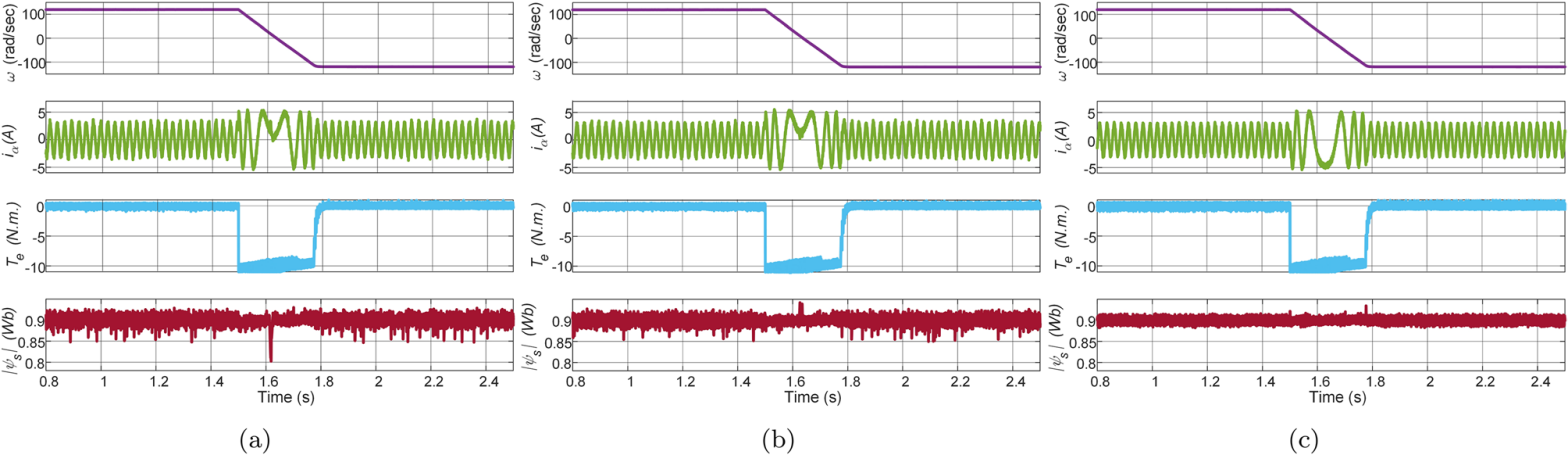
Experimental response of speed reversal test of motor under no-load condition for speed. (**a**) Con-MPTC, (**b**) Vik-MPTC, (**c**) Ent-MPTC.

The transient response of Con-MPTC, Vik-MPTC, and proposed Ent-MPTC under speed reversal conditions at a rated speed of $120\, \mathrm {rad/s}$ without load was obtained on the IM drive with the current model estimator (CM). The response of the speed reversal test is depicted in Fig. 7. Each plot contains reference speed, phase $\text{a}$ stator current, electromagnetic torque, and stator flux magnitude. The speed reverses from $120\,\mathrm {rad/s}$ at time $1.5\, \text{sec}$ to $-120\, \mathrm {rad/s}$ at time $1.8\, \text{s}$. The stator reference flux is assumed to be constant at a rated value of $0.9\, \text{Wb}$. It can be observed from comparing these results that the Ent-MPTC provides comparable performance to Con-MPTC and Vik-MPTC. Since Con-MPTC operates on a nominal static weighting factor and Vik-MPTC tunes the weighting factor online, the proposed method outperforms the flux regulation. The current distortion can be observed in Con-PTC and Vik-MPTC compared to Ent-MPTC.

**Figure 8:**
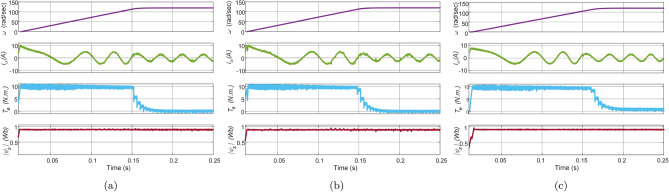
Experiment result of no-load response of IM drive at speed of $120\,\mathrm {rad/s}$. (**a**) Con-MPTC, (**b**) Vik-MPTC, (**c**) Ent-MPTC.

The experimental results of the no-load test under reference speed of $120\, \mathrm {rad/s}$ are shown in Fig. 8. The no-load test of Con-MPTC, Vik-MPTC, and Ent-MPTC shows that the proposed weighting factor tuning method performs satisfactorily as compared to the other two techniques.

### Steady state response

**Figure 9:**
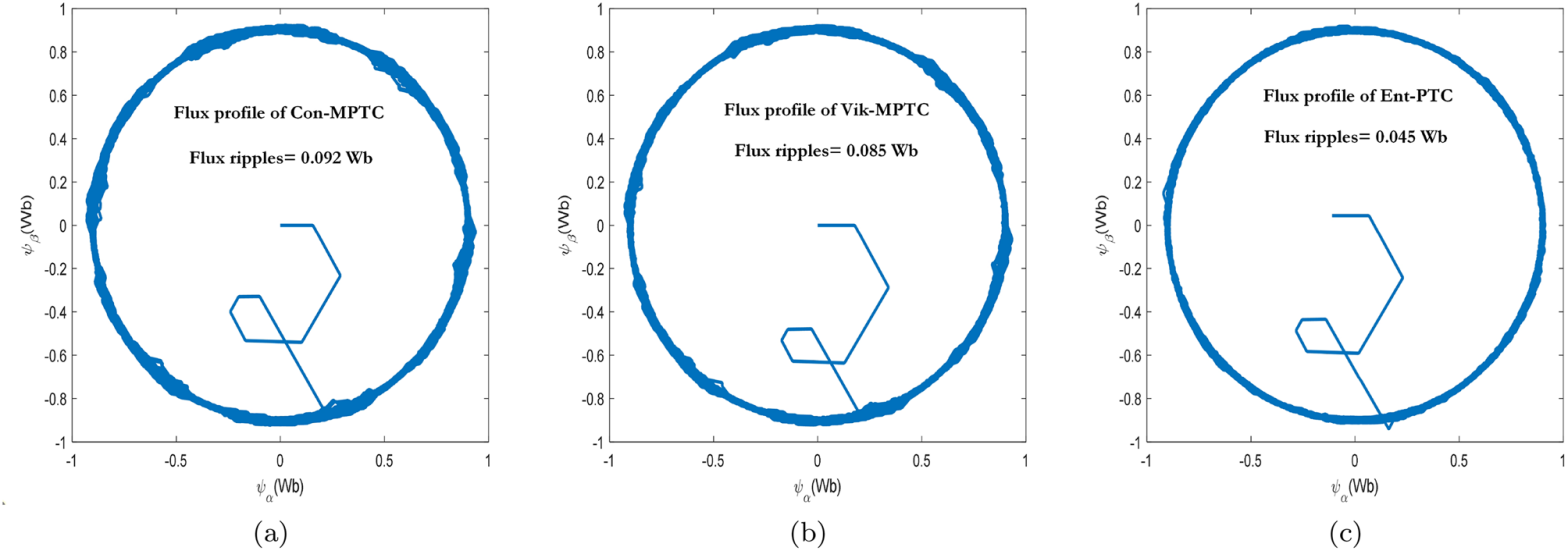
Flux trajectories under steady state condition of IM drive. (**a**) Con-MPTC, (**b**) Vik-MPTC, (**c**) Ent-MPTC.

**Figure 10:**
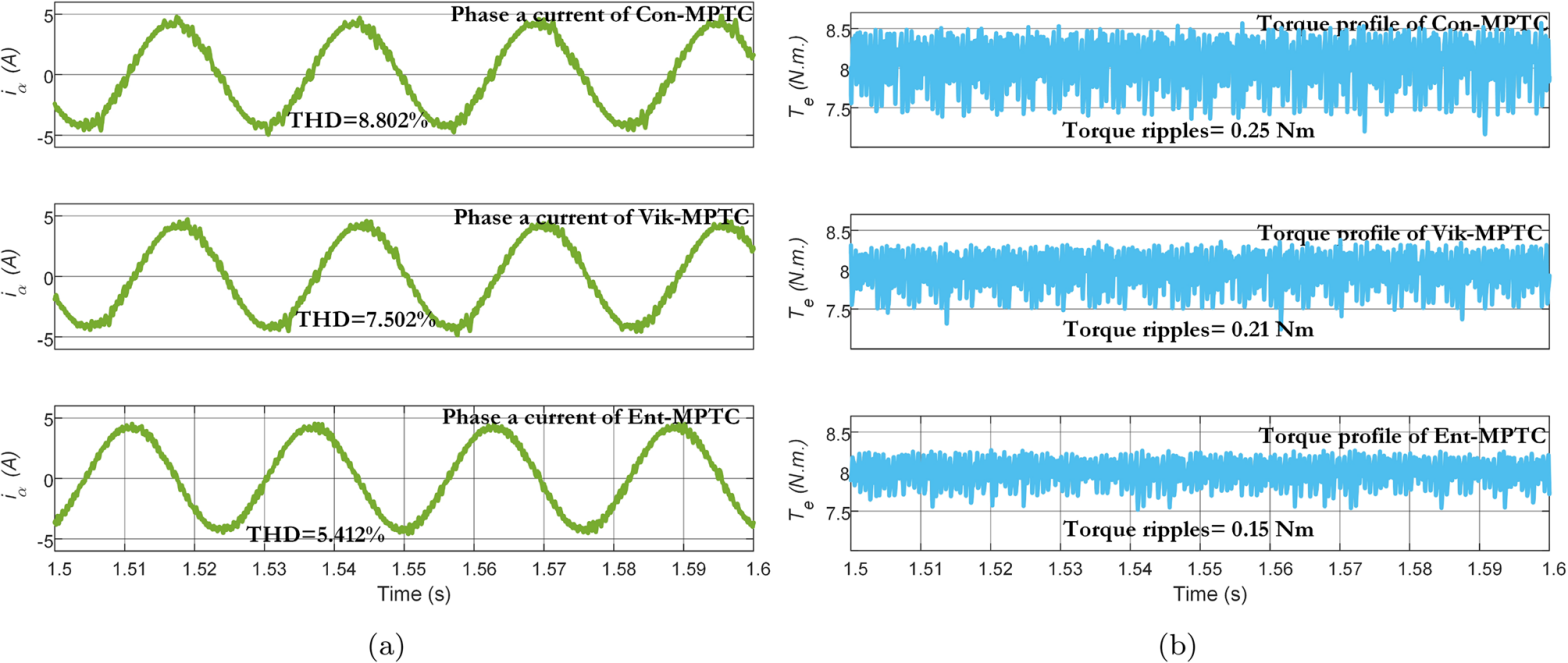
Torque and current profile under steady state condition for Con-MPTC, Vik-MPTC and Ent-MPTC. (**a**) Current profile, (**b**) Torque profile.

The steady-state response of Con-MPTC, Vik-MPTC, and Ent-MPTC was recorded at a rated speed of $120\, \mathrm {rad/s}$, and a load of $8\, \text{Nm}$ was applied to the drive. Figure 9 presents the flux trajectories under steady-state conditions. It can be observed from the trajectories of the three methods that the average flux ripples are $0.092\,\text{Wb}$ and $0.085\,\text{Wb}$ for Con-MPTC and Vik-MPTC, respectively. Whereas, Ent-MPTC has average flux ripples of $0.045\,\text{Wb}$, representing an almost ${50\%}$ reduction in flux ripples compared with Con-MPTC and Vik-MPTC. The steady-state performance of the three given techniques is compared according to THD and average torque ripples. The results of phase a current and torque profiles of three techniques are given in Fig. 10. It is evident from the current profile that Ent-MPTC shows THD=${5.41\%}$ as compared with ${8.80\%}$ and ${7.50\%}$ in Con-MPTC and Vik-MPTC, respectively. The average torque ripples of the three techniques are compared in Fig. 10b. The reduction in average torque ripples validates the effectiveness of the proposed method. A ${40\%}$ reduction is achieved in Ent-MPTC compared with Con-MPTC, whereas a ${28\%}$ reduction in average torque ripples compared with Vik-MPTC. The proposed method’s improved average torque ripples, average flux ripples, and THD prove the effectiveness over the Con-MPTC and Vik-MPTC.

To compare the steady-state performance of the proposed method with Conv-MPTC and Vik-MPTC at nominal load torque, the drive was tested at different speeds of 46 rad/s, 96 rad/s, and 120 rad/s, respectively. At each speed, average torque ripple, average flux ripple, switching frequency and THD are recorded and presented in Table 4. These results show that the proposed method outperformed the other two methods in terms of all performance metrics. This table validates the effectiveness of the proposed method with improved performance due to dynamic online tuning of the weighting factor.

**Table 4:** Performance comparison of proposed method with Con-MPTC, Vik-MPTC and Ent-MPTC.

Speed (rad/s)	MPTC method	$T_{rip}$ (Nm)	$Flux_{rip}$ (Wb)	$f_{sw}(kHz)$	THD ($\%$)
46	Conv-MPTC	0.38	0.099	7.378	11.8
	Vik-MPTC	0.31	0.089	7.182	10.78
	Emt-MPTC	0.25	0.081	6.572	9.12
96	Conv-MPTC	0.35	0.098	11.420	9.18
	Vik-MPTC	0.30	0.091	11.280	9.02
	Ent-MPTC	0.21	0.071	10.170	4.41
120	Conv-MPTC	0.25	0.092	11.860	8.90
	Vik-MPTC	0.21	0.085	11.800	7.45
	Ent-MPTC	0.15	0.045	11.130	4.06

**Figure 11:**
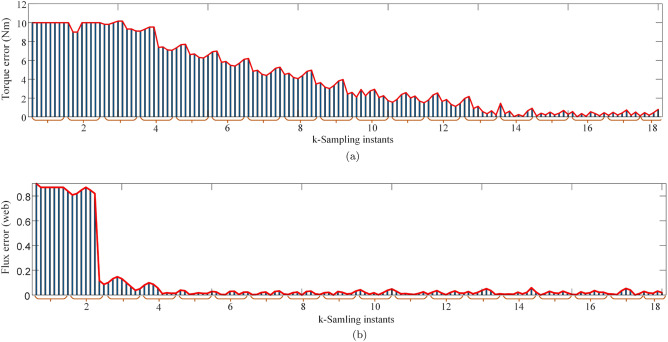
Minimum torque and flux errors under $120\, \mathrm {rad/s}$ for k-sampling instants. (**a**) Torque error response, (**b**) Flux error response.

**Figure 12:**
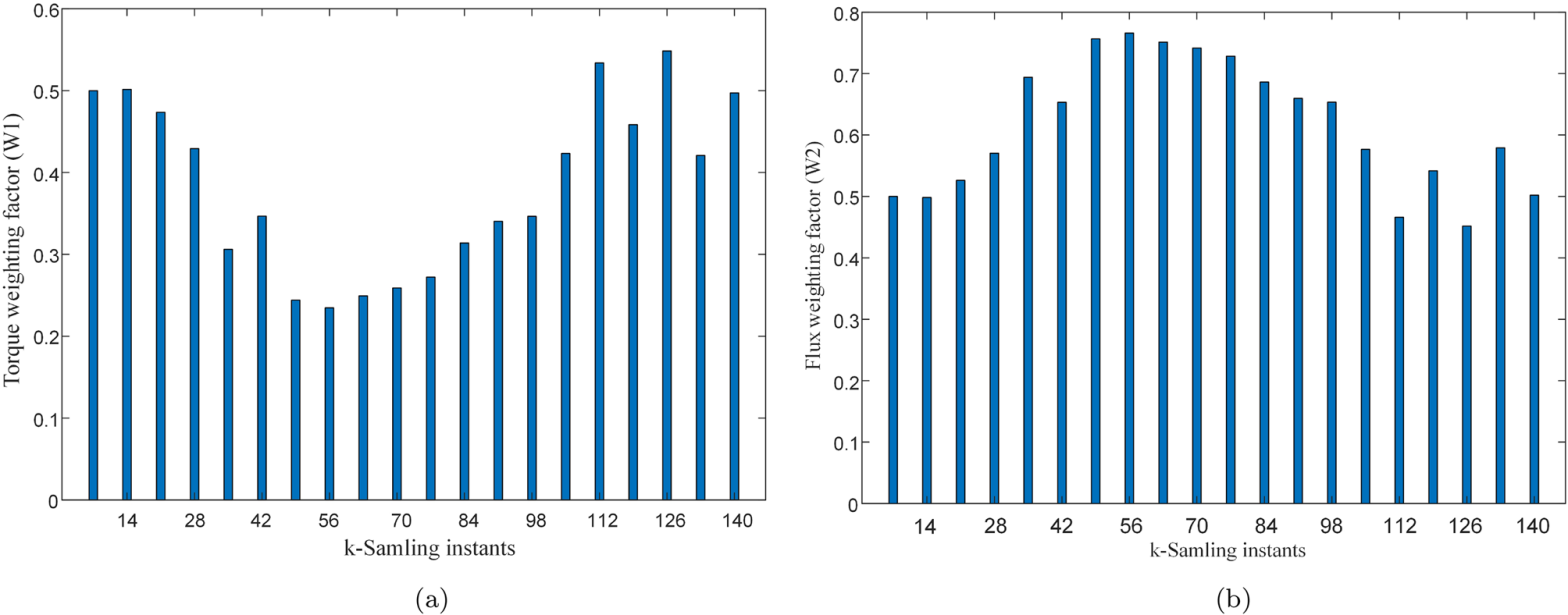
Weights at k sampling instant. (**a**) weighting factor for torque, (**b**) weighting factor for flux.

It is also important to show how the weights are optimized by the entropy technique and how the errors are reduced with time. Figure 11a,b represent values of the torque and flux errors at each sampling instant during steady state operation of the drive. It can be observed from the figure that errors remain higher during the initial instants of time but gradually decrease as the time progresses. The weighting factors are tuned at each sampling instant interval with higher priority given to the controlled variable with relatively higher error. Initially, both control variables are given almost equal relative importance and weights are almost equal to 0.5. As the controller reduces the errors, the weights keep on changing for improving the performance. These weights are captured and shown in Fig. 12a,b. It can also be obsered that as the torque error reduces so does its weight to put more emphasis on flux error hence confirming the dynamic tuning of weights.

### Computational time

**Figure 13:**
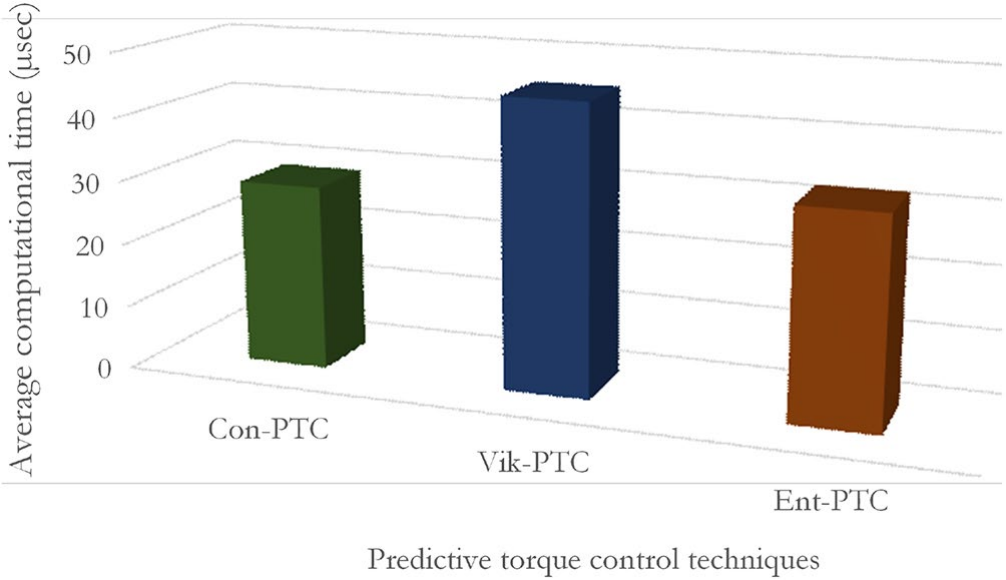
Average computational time of Con-MPTC, Vik-MPTC and Ent-MPTC.

The computation time is recorded to assess the average execution time of three methods on the DS1104 controller board. The average computational time is divided into the measurement of control variables, prediction of flux and torque, determination of weighting factor, and last is cost function optimization. The Con-MPTC takes less computational time than the other two techniques due to the nominal weighting factor. The average computational burden of Con-MPTC, Vik-MPTC, and Ent-MPTC is given in the Fig. 13.

It is evident from the figure that Con-MPTC, Vik-MPTC, and Ent-MPTC take average computational time of $29.05\, \upmu \text{sec}$, $45.25\, \upmu \text{sec}$, and $32.5\, \upmu \text{sec}$, respectively. Con-MPTC takes minimum computational time due to the use of nominal weighting factor, and Ent-MPTC takes $\mathrm {28\%}$ less average computational time as compared to Vik-MPTC because of a simple algorithm to determine weighting factors.

### Loaded response

**Figure 14:**
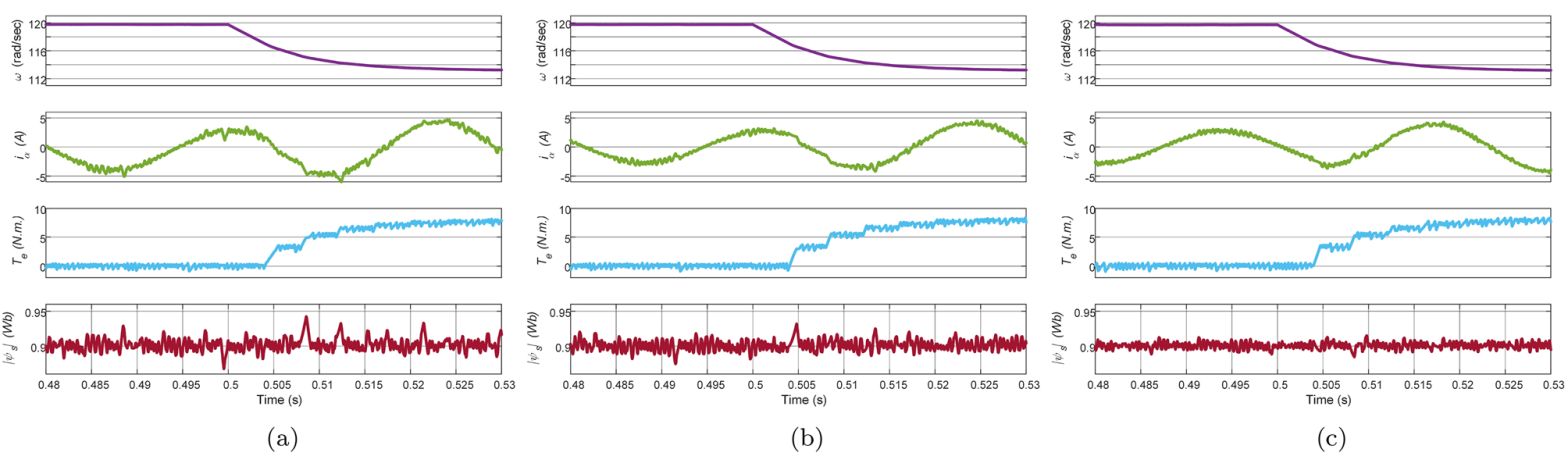
Loaded response of IM drive at speed of $120\,\mathrm {rad/s}$ and load of $8\,\text{Nm}$ applied at $\text{t}=0.5\,\text{sec}$. (**a**) Con-MPTC, (**b**) Vik-MPTC, (**c**) Ent-MPTC.

In this test, the IM drive is loaded with a load torque of $8\,\text{Nm}$ at $\text{t}=0.5\,\text{sec}$ at a speed of $120\,\mathrm {rad/sec}$. The waveform of reference speed, phase $\text{a}$ stator current, electromagnetic torque, and stator flux are depicted in Fig. 14. It is evident from the figure that all the methods have similar transient response with a maximum current of $5\,\text{A}$. However, torque and flux ripples are significant in Con-MPTC and Vik-MPTC as compared to Ent-MPTC. In the starting, Con-MPTC and Vik-MPTC have THD of 10.47% and 10.04%, respectively. Similarly, both techniques have average flux ripples of 0.09 and $0.05\,\text{Wb}$ . However, in the case of Ent-MPTC, it has a THD of 6.32% and $0.02\,\text{Wb}$ average flux ripple. The speed reduction is also observed in all the techniques due to the IM drive’s loading effect.

The transient performance of the proposed method in the loaded condition is comparable with conventional and Vik-MPTC techniques and a slight improvement is also observed in terms of average flux ripples. To overcome the loading effects on the mechanical speed, a disturbance rejection technique can be incorporated in the outer speed loop.

### Parameter variation

The performance in steady-state conditions is negatively impacted by the parameter variations in the system model, given that the MPC methodology depends on the explicit system model to achieve control objectives through prediction. In the subsequent part, the robustness performance of Con-MPTC, Vik-MPTC, and Ent-MPTC, has been evaluated and compared by varying the stator resistance $\text{R}_{\text{s}}$, rotor resistance $\text{R}_{\text{r}}$, and mutual inductance $\text{L}_{\text{m}}$. The variation in $\text{R}_{\text{r}}$ is very significant as it directly affects the rotor time constant $\tau _{\text{r}}=\frac {Lr}{\text{R}_{\text{r}}}$, which in turn has a negative impact on the accuracy of the predictions. The variation in $\tau _{\text{r}}$ also deteriorates the performance of rotor speed controller.

**Figure 15:**
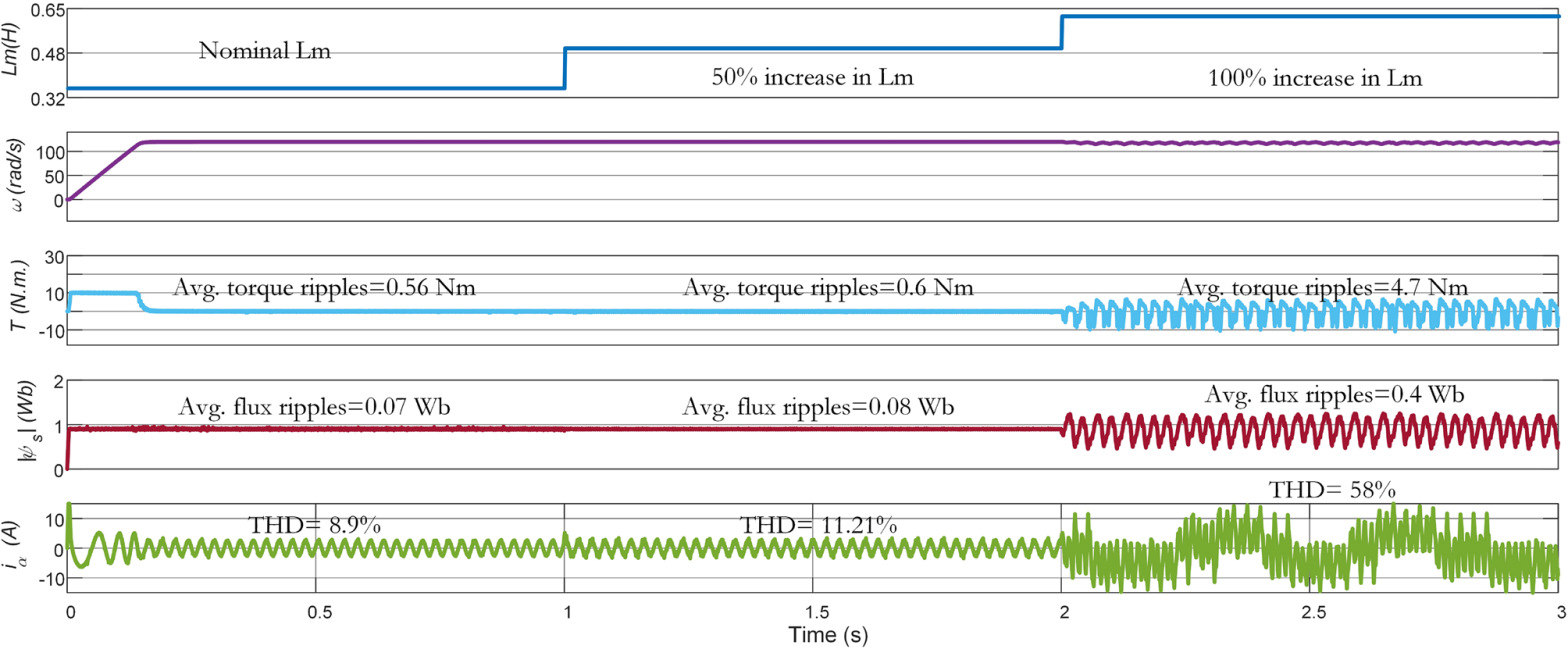
Experimental results of mutual inductance variation from nominal to $\mathrm {50\%}$ and $\mathrm {100\%}$ for Con-MPTC.

**Figure 16:**
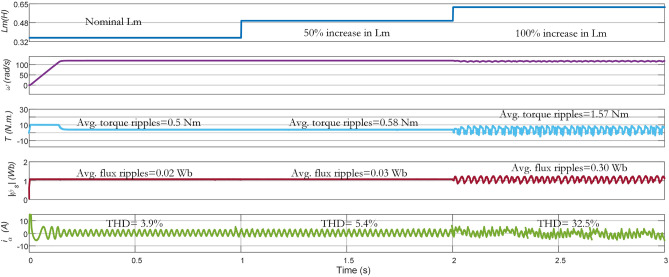
Experimental results of mutual inductance variation from nominal to $\mathrm {50\%}$ and $\mathrm {100\%}$ for Vik-MPTC.

In this work, the performance of the three methods is compared under (i) the nominal value of the parameters (ii) 1.5 times of nominal value (iii) 2 times of the nominal value. The average torque ripple, flux ripple, and THD are recorded under the parameter variations. In Fig. 15, results of $\text{L}_{\text{m}}$ variation are given for conventional PTC. At nominal $\text{L}_{\text{m}}$, the average torque ripple, average flux ripple, and THD are $0.56\,\text{Nm}$, $0.07\,\text{Wb}$, and 8.9%, respectively. As it can be noted in the figure, increasing $\text{L}_{\text{m}}$ from its nominal value to 1.5 (times $\mathrm {50\%}$ increase) and $\text{2}$ times ($\mathrm {100\%}$ increase), negatively impacts the performance of the controller. When the increase in $\text{L}_{\text{m}}$ is $\mathrm {100\%}$, the performance becomes very poor with $4.7\,\text{Nm}$ average torque ripple, $0.4\,\text{Wb}$ average flux ripple and $\mathrm {58\%}$ THD. The reason behind this deterioration is the use of fixed weighting factor in the Con-MPTC.

**Figure 17:**
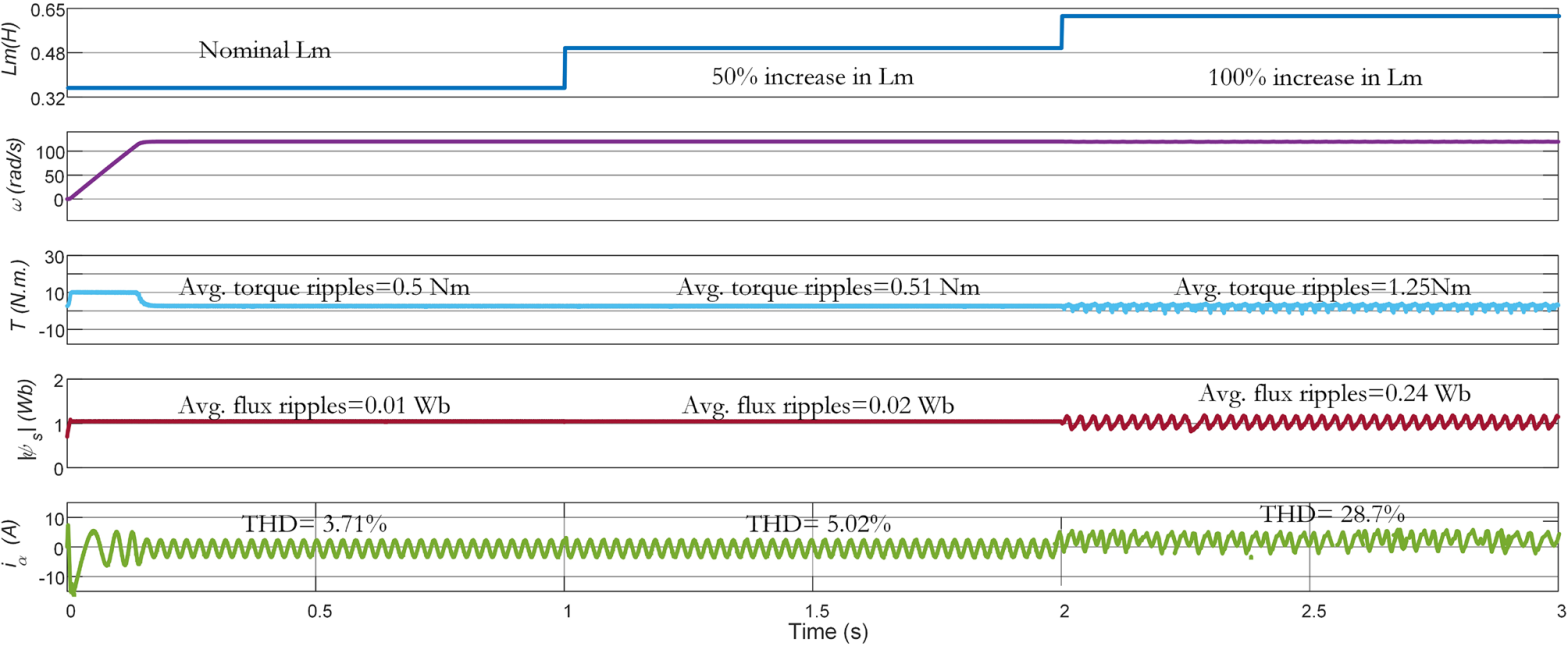
Experimental results of mutual inductnace variation from nominal to $\mathrm {50\%}$ and $\mathrm {100\%}$ for Ent-MPTC.

Impacts of $L_m$ variations on the performance of Vik-MPTC are presented in Fig. 16. It can be seen from the obtained results that Vik-MPTC has $0.5\,\text{Nm}$ average torque ripple, $0.02\,\text{Wb}$ average flux ripple and 3.9 THD at nominal $\text{L}_{\text{m}}$. Whereas, after $\text{L}_{\text{m}}$ is changed beyond its nominal value, the performance of the controller deteriorates. For $\mathrm {100\%}$ increase in $\text{L}_{\text{m}}$, average torque ripple is $1.57\,\text{Nm}$, average flux ripple is $0.3\,\text{Wb}$ and THD is 32.5%. Similarly Fig. 17 represents the effects of the variation of $\text{L}_{\text{m}}$ on the performance of the drive. Ent-MPTC provides lower THD as $L_m$ varies from nominal value to $\mathrm {100\%}$ increase as compared with Con-MPTC and Vik-MPTC. The proposed Ent-MPTC provides better control over the $\mathrm {100\%}$ increase of $\text{L}_{\text{m}}$. It can be concluded that variation in $\text{L}_{\text{m}}$ in Ent-MPTC produces lower disturbance than Con-PTC and Vik-MPTC. This is due to a simple algorithm to determine the weighting factor and the selection of optimal weights of control objectives in the proposed method.

**Figure 18:**
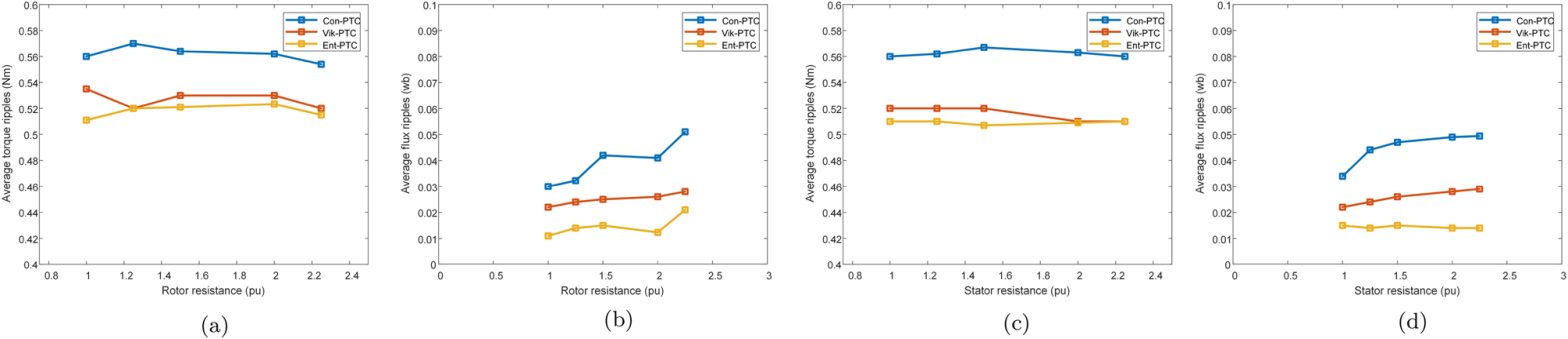
Effect of stator and rotor resistance variation on average torque and flux ripples. (**a**) Torque ripples under $\text{R}_{\text{r}}$ variations, (**b**) Flux ripples under $\text{R}_{\text{r}}$ variations, (**c**) Torque ripples under $\text{R}_{\text{s}}$ variations, (**d**) Flux ripples under $\text{R}_{\text{s}}$ variations.

To see the effects of variation in $\text{R}_{\text{r}}$ and $\text{R}_{\text{s}}$ on the performance of different controllers, the drive was tested under full load and rated speed. The parameters were changed from their nominal values to 2.2 times the nominal values. The results of these tests are presented in Fig. 18. From the presented results, it can be seen that the variations have not much effect on the performance of three controllers. The torque and flux ripples do not change to a greater extent while these parameters vary. However, the ripples remain lowest for Ent-MPTC as compared to other controllers.

Figure 18a,b represents effect of rotor resistance over average torque and average flux ripples. The result was compared to Con-MPTC, Vik-MPTC and Ent-MPTC. Although all the methods exhibits lower effect on average torque and flux ripples, however Ent-MPTC presents lower torque and flux ripples as compared with Con-MPTC and Vik-MPTC. Similarly Fig. 18c,d shows the performance of the derive under variation of stator resistance. It can be observed from figure that the Ent-MPTC outperform over the Con-MPTC and Vik-MPTC and variation in $\text{R}_{\text{s}}$ does not effect the average flux ripples and it also offers lower average flux ripples as compared to Con-MPTC and Vik-MPTC. These results validate the robustness of the proposed method.

### Load and speed variations

**Figure 19:**
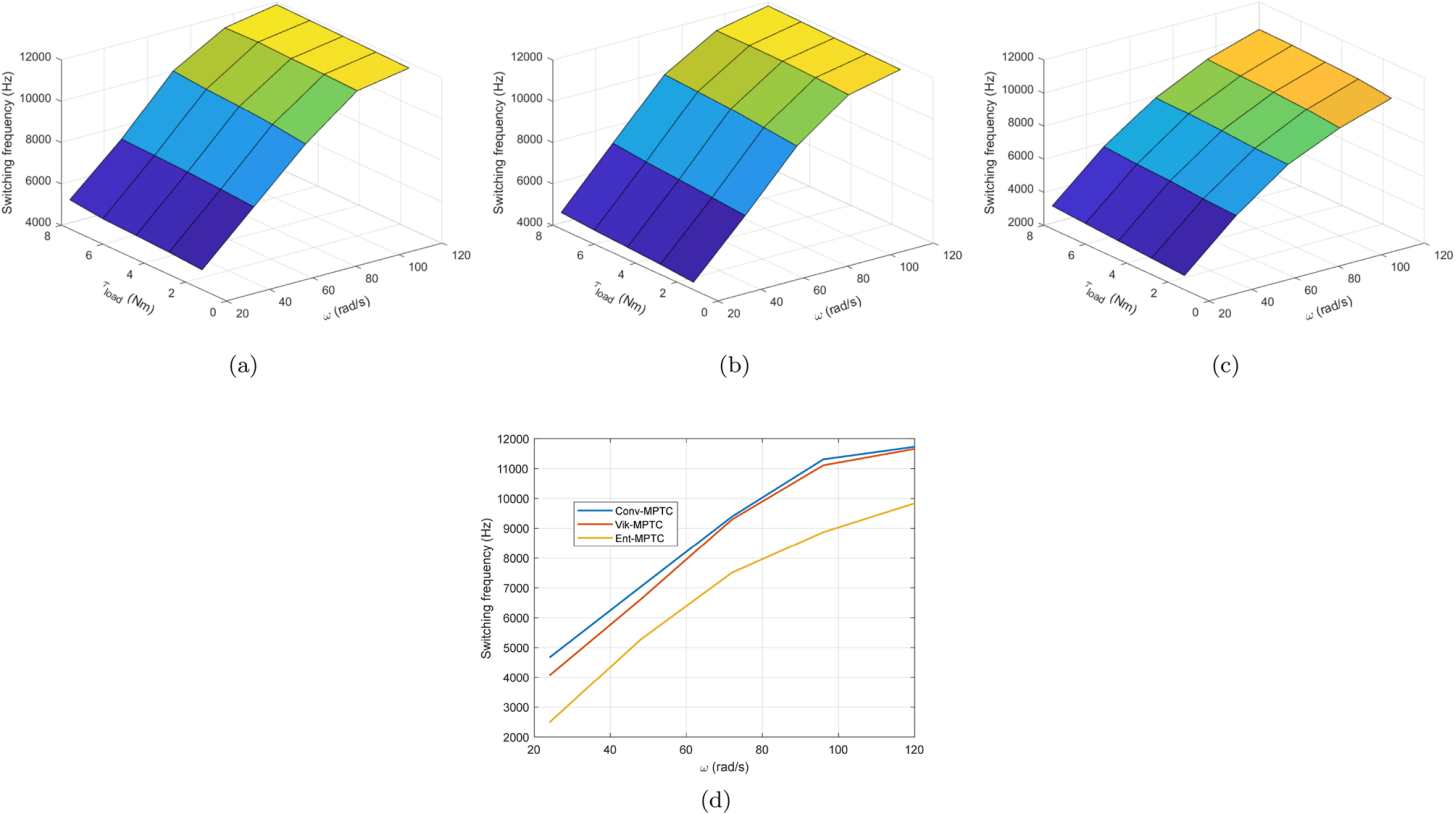
Switching frequency with variation of load torque and speed. (**a**) Con-MPTC, (**b**) Vik-MPTC, (**c**) Ent-MPTC, (**d**) Cross-sectional comparison $\text{f}_{\text{sw}}$ of three methods at $\text{T}_{\text{L}=1.6 \text{Nm}}$.

**Figure 20:**
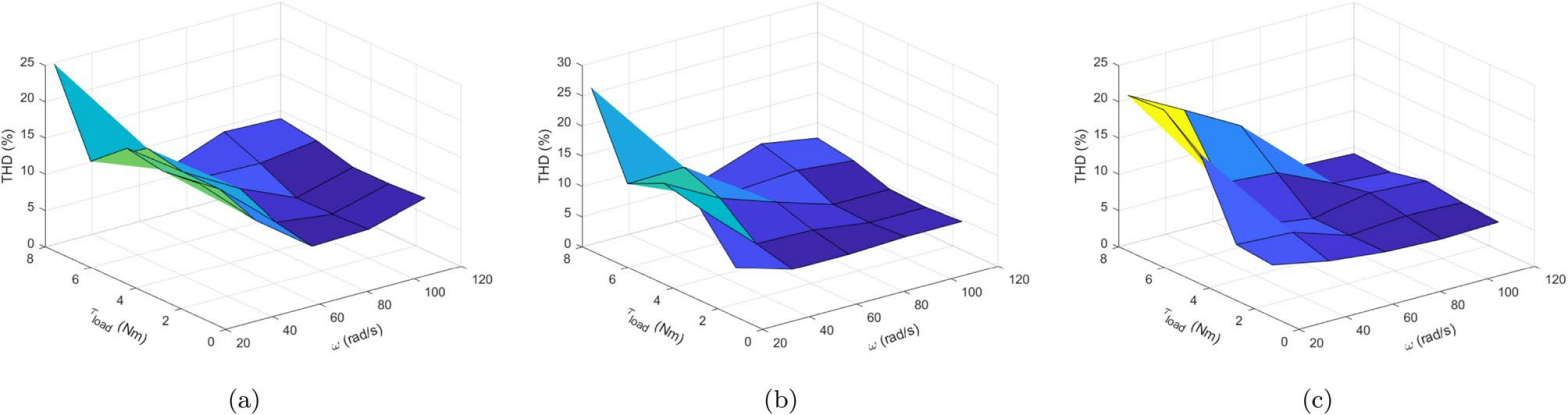
Total harmonic distortion (THD) with variation of load torque and speed. (**a**) Con-MPTC, (**b**) Vik-MPTC, (**c**) Ent-MPTC.

The effect of load torque and speed variation on the system’s average switching frequency and THD are observed and depicted in Figs. 19 and 20, respectively. The speed and load torque varies from zero to rated value in steps of $\mathrm {20\%}$ whereas speed is gradually increased in steps of $20\, \mathrm {rad/s}$. It is concluded from the figure that three of the PTC methods have almost the similar switching frequency patterns. However, Ent-MPTC shows lower switching frequency at high speeds and higher loads. In Ent-MPTC, the switching frequency varies up to $10\,\text{kHz}$; however, it is up to $12\,\text{kHz}$ in Con-MPTC and Vik-MPTC. The cross section of Fig. 19a–c is shown in (d). This cross section is taken at load torque of $1.6\, \text{Nm}$ for Con-MPTC, Vik-MPTC and Ent-MPTC. It can be observed from the figure that proposed method works on lower switching frequency compared with Con-MPTC and Vik-MPTC. Similar to switching frequency, the effect of load torque and speed variation is observed on THD. THD of Con-PTC at full load and low speed is $\mathrm {25\%}$, and at the same position, Vik-MPTC and Ent-MPTC have $\mathrm {26\%}$ and $\mathrm {20\%}$, respectively. The THD of Con-MPTC varies from a maximum of $\mathrm {24\%}$ to a minimum of 8.9% with the variation of speed and load, as depicted in the figure. Whereas in the case of Vik-MPTC, THD varies from 25 to 7.46%, and higher THD fluctuations can be seen in Con-MPTC and Vik-MPTC. In Ent-MPTC, THD varies from a maximum of $\mathrm {20\%}$ to its minimum value of 4.06% at full load and speed region. Furthermore, Ent-MPTC has minimum fluctuations in THD over a wide load and speed range. That is due to the optimal selection of the weighting factor in Ent-MPTC.

## Conclusion

Weighting factor selection has remained a challenging and complex task in finite set model predictive control applications. In this paper, a multi-criteria-decision-making (MCDM) based entropy method is used to determine the optimal weighting factor. The effectiveness of the proposed method is verified by an experimental setup based on dSpace dS1104 controller. The experimental results of the entropy method are compared with conventional MPTC and another MCDM-based technique known as the VIKOR method. The superiority of the proposed method is validated under steady state operation by $\mathrm {38\%}$ reduction in THD as compared with conventional MPTC and $\mathrm {27\%}$ reduction in THD as compared with Vik-MPTC. The average flux ripples reduced upto and $\mathrm {51\%}$ compared with conventional MPTC and $\mathrm {47\%}$ reduction with Vik-MPTC. Similarly, a $\mathrm {40\%}$ reduction in average torque ripple was observed as compared with Conv-MPTC, and a $\mathrm {28\%}$ reduction was recorded as compared with Vik-MPTC. Moreover, the proposed method reduces the computational burden up to $\mathrm {28\%}$ compared to Vik-MPTC. The proposed method also performed better in different speed ranges and showed efficient dynamic and steady state response as compared to Conv-MPTC and Vik-MPTC. Additionally, it has the advantage of robustness against Con-MPTC and Vik-MPTC by parameter variation of $\text{L}_{\text{m}}$, $\text{R}_{\text{r}}$ and $\text{R}_{\text{r}}$. It is concluded that most online weighting factor tuning techniques pose a a higher computational burden, but the proposed technique can incorporate more than two control objectives into the cost function without increasing the computational burden due to its simple algorithm.

## Data Availability

Data generated or analyzed during this study is provided within this manuscript.
